# LDHB contributes to the regulation of lactate levels and basal insulin secretion in human pancreatic β cells

**DOI:** 10.1016/j.celrep.2024.114047

**Published:** 2024-04-11

**Authors:** Federica Cuozzo, Katrina Viloria, Ali H. Shilleh, Daniela Nasteska, Charlotte Frazer-Morris, Jason Tong, Zicong Jiao, Adam Boufersaoui, Bryan Marzullo, Daniel B. Rosoff, Hannah R. Smith, Caroline Bonner, Julie Kerr-Conte, Francois Pattou, Rita Nano, Lorenzo Piemonti, Paul R.V. Johnson, Rebecca Spiers, Jennie Roberts, Gareth G. Lavery, Anne Clark, Carlo D.L. Ceresa, David W. Ray, Leanne Hodson, Amy P. Davies, Guy A. Rutter, Masaya Oshima, Raphaël Scharfmann, Matthew J. Merrins, Ildem Akerman, Daniel A. Tennant, Christian Ludwig, David J. Hodson

**Affiliations:** 1 Institute of Metabolism and Systems Research (IMSR) and Centre of Membrane Proteins and Receptors (COMPARE), University of Birmingham, Birmingham, UK; 2 Oxford Centre for Diabetes, Endocrinology and Metabolism (OCDEM), NIHR Oxford Biomedical Research Centre, Churchill Hospital, Radcliffe Department of Medicine, University of Oxford, Oxford, UK; 3 Geneplus-Beijing, Changping District, Beijing 102206, China; 4 Oxford Kavli Centre for Nanoscience Discovery, University of Oxford, Oxford, UK; 5 University of Lille, Institut National de la Santéet de la Recherche Médicale (INSERM), Centre Hospitalier Universitaire de Lille (CHU Lille), Institute Pasteur Lille, U1190-European Genomic Institute for Diabetes (EGID), F59000 Lille, France; 6 San Raffaele Diabetes Research Institute, IRCCS Ospedale San Raffaele, Milan, Italy; 7 Vita-Salute San Raffaele University, Milan, Italy; 8 Nuffield Department of Surgical Sciences, University of Oxford, John Radcliffe Hospital, Oxford, UK; 9 Centre for Systems Health and Integrated Metabolic Research (SHiMR), Department of Biosciences, School of Science and Technology, Nottingham Trent University, Nottingham, UK; 10 Section of Cell Biology and Functional Genomics, Division of Diabetes, Endocrinology and Metabolism, Department of Metabolism, Digestion and Reproduction, Imperial College London, London, UK; 11 CHUM Research Centre and Faculty of Medicine, University of Montreal, Montreal, QC, Canada; 12 Lee Kong Chian School of Medicine, Nanyang Technological University, Singapore, Singapore; 13 Université Paris Cité, Institut Cochin, INSERM U1016, CNRS UMR 8104, 75014 Paris, France; 14 Department of Medicine, Division of Endocrinology, Diabetes, and Metabolism, University of Wisconsin-Madison, Madison, WI 53705, USA; 15 William S. Middleton Memorial Veterans Hospital, Madison, WI 53705, USA; 16 These authors contributed equally; 17 Lead contact

## Abstract

Using ^13^C_6_ glucose labeling coupled to gas chromatography-mass spectrometry and 2D ^1^H-^13^C heteronuclear single quantum coherence NMR spectroscopy, we have obtained a comparative high-resolution map of glucose fate underpinning β cell function. In both mouse and human islets, the contribution of glucose to the tricarboxylic acid (TCA) cycle is similar. Pyruvate fueling of the TCA cycle is primarily mediated by the activity of pyruvate dehydrogenase, with lower flux through pyruvate carboxylase. While the conversion of pyruvate to lactate by lactate dehydrogenase (LDH) can be detected in islets of both species, lactate accumulation is 6-fold higher in human islets. Human islets express LDH, with low-moderate LDHA expression and β cell-specific LDHB expression. LDHB inhibition amplifies LDHA-dependent lactate generation in mouse and human β cells and increases basal insulin release. Lastly, *cis*-instrument Mendelian randomization shows that low LDHB expression levels correlate with elevated fasting insulin in humans. Thus, LDHB limits lactate generation in β cells to maintain appropriate insulin release.

## In brief

Cuozzo et al. show that glucose-stimulated rodent and human islets generate lactate. Transcriptomic, imaging, and Mendelian randomization analyses reveal that LDHB restrains β cell lactate production and contributes to basal/fasting insulin release. LDHB expression and thus regulated lactate generation might reflect a key mechanism underlying β cell metabolism and function.

## Graphical Abstract

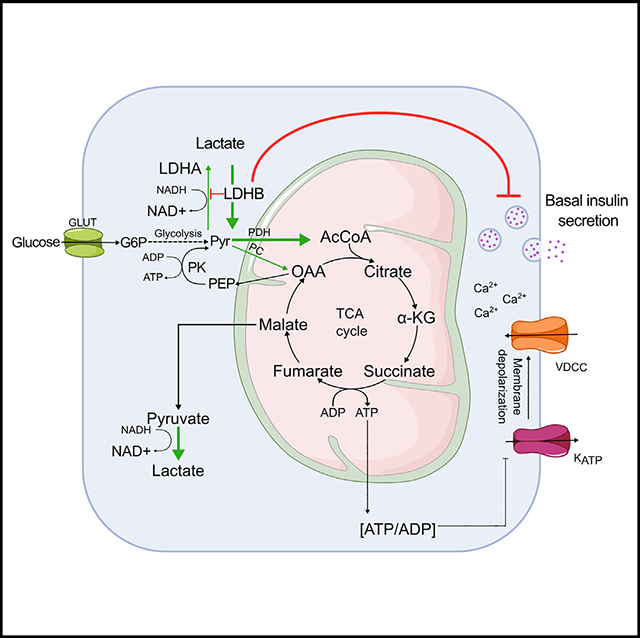

## INTRODUCTION

Following a rise in glycemia, glucose enters the pancreatic β cell through facilitated transport via low-affinity glucose transporters (GLUT1 and GLUT2 in humans and rodents, respectively).^1,2^ Glucose is then phosphorylated by glucokinase (GK), leading to the closure of ATP-sensitive potassium (K_ATP_) channels (reviewed in Rorsman and Ashcroft and Rutter et al.^3,4^). The increase in membrane voltage drives Ca^2+^ flux through voltage-dependent Ca^2+^ channels,^3^ which, together with amplifying signals,^4–6^ evokes insulin granule exocytosis. Direct conversion of pyruvate to lactate is suppressed in the β cell due to low levels of lactate dehydrogenase A (*LDHA*),^7–10^ ensuring that the majority of pyruvate enters the tricarboxylic acid (TCA) cycle.

Recent studies have challenged the canonical view of β cell metabolism by showing that ATP/ADP generation might be compartmentalized. K_ATP_ channels are locally regulated by a membrane-associated glycolytic metabolon that is assisted by the mitochondrial phosphoenolpyruvate (PEP) cycle.^11–13^ The glucose-dependent rise in the ATP/ADP ratio raises mitochondrial voltage to stall oxidative phosphorylation and the TCA cycle, activating anaplerotic flux through pyruvate carboxylase (PC) and the PEP cycle, facilitating ATP production by pyruvate kinase (PK) until K_ATP_ channels are closed. Following membrane depolarization, the rise in ADP supports a highly oxidative state that depends on high TCA cycle flux and pyruvate consumption by pyruvate dehydrogenase (PDH), which supports oxidative phosphorylation and sustained secretion.^11,12,14^

Alongside compartmentalized ATP/ADP generation, anaplerotic metabolism serves as an important source of coupling or amplifying factors for glucose-stimulated insulin secretion.^15–17^ For example, PC directs pyruvate entry into the TCA cycle, which feeds isocitrate into isocitrate dehydrogenase 1 (IDH1) to support 2-ketoglutarate (2-KG)/NADPH generation and SENP1 activation.^6,18^ This reaction is further supported by metabolism of glutamine by the reductive TCA cycle.^19^

Despite the clear importance of metabolism for β cell insulin release and phenotype, we are still lacking a high-resolution, integrated view of β cell glucose fate across species. Most data using glucose tracing and gas chromatography-mass spectrometry (GC-MS)/NMR spectroscopy has been derived from insulinoma cell lines, which provide the requisite cell mass for metabolite detection/annotation. However, insulinoma cell lines have to balance the need for insulin secretion with proliferation, an energy-consuming process,^15,17,20–23^ and fail to display normal cell heterogeneity known to influence metabolism.^24–26^ Recent studies have used metabolic tracing to compare stem cell-derived β cell and human islet metabolism, highlighting differences in glucose metabolism, metabolite trafficking, and fuel sensitivity.^27,28^ However, these studies did not combine GC-MS/liquid chromatography-mass spectrometry (LC-MS) with NMR for TCA metabolite annotation, nor cross-compare human and mouse islets.

In the present study, we combine GC-MS-based ^13^C_6_ glucose tracing with the resolution of 2D ^1^H-^13^C HSQC NMR multiplet analysis to map glucose fate in islets with high sensitivity. By applying this dual approach to human and mouse samples, we are able to provide a detailed cross-species depiction of glucose metabolism.

## RESULTS

### Glucose contribution to the TCA cycle in human and mouse islets

**Figure 1.**
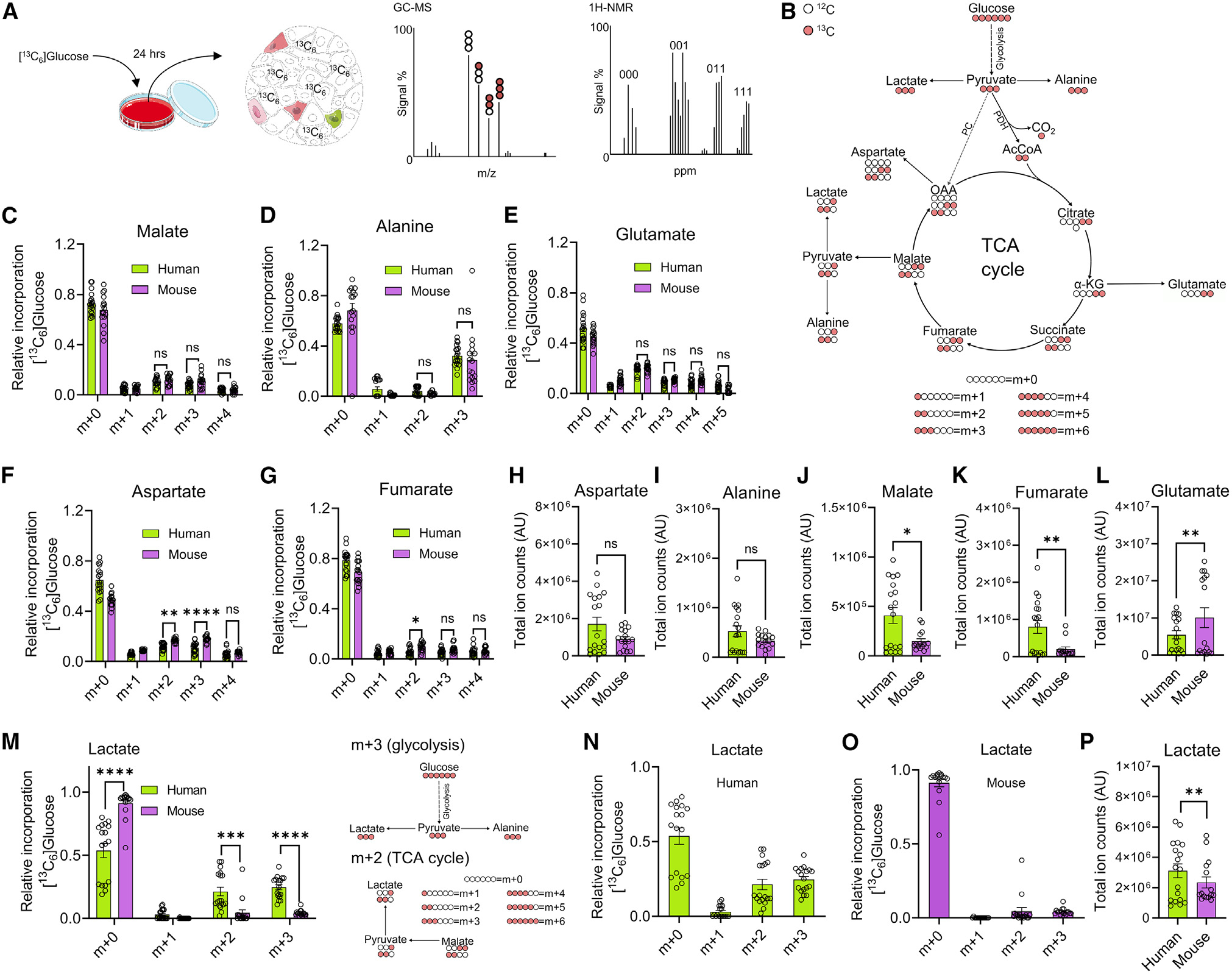
MID analysis of glucose fate in human and mouse islets (A) GC-MS and ^1^H-NMR-based ^13^C_6_ glucose tracing in primary islets. B) MID analysis of ^13^C_6_ glucose-tracing data. (C–E) MID analysis showing similar incorporation of ^13^C from ^13^C_6_ glucose into malate (C), alanine (D), and glutamate (E) in human and mouse islets. (F and G) MID analysis showing increased incorporation of ^13^C from ^13^C_6_ glucose into m + 2 and m + 3 aspartate (F), and m + 2 fumarate (G) in mouse compared to human islets. (H and I) Total amount of extracted aspartate (H) and alanine (I) is similar in human and mouse islets. (J–L) Total amount of extracted malate (J) and fumarate (K) is decreased in mouse relative to human islets, whereas glutamate (L) is increased. (M–O) MID analysis shows detectable glucose incorporation into m + 2 (TCA cycle) and m + 3 (pyruvate conversion) lactate, with more accumulation in human (M and N) versus mouse (M and O) islets (*n* = 18 independent replicates from nine human donors; *n* = 10 islet preparations from fifteen animals) (N and O show same data as M, but as separate graphs for clarity). (P) Total lactate generation is higher in human compared to mouse islets (*n* = 18 independent replicates from nine human donors; *n* = 10 islet preparations from fifteen animals). (C–G and M) Analyzed using two-way ANOVA and Sidak’s *post hoc* test. (H–L and P) Analyzed using Welch’s test. Bar graphs show individual datapoints and mean ± SEM. ns, non-significant; *p < 0.05; **p < 0.01; ***p < 0.001; ****p < 0.0001. AU, arbitrary unit.

Mouse and human islets were incubated overnight with ^13^C_6_ glucose prior to metabolite extraction, GC-MS and 2D ^1^H,^13^C HSQC-NMR spectroscopy (Figure 1A), and mass isotopologue distribution (MID) analysis (Figure 1B). Suggesting a similar progression of glycolysis and the TCA cycle, glucose incorporation into glycerol-3-phosphate (G-3-P) (Figure S1A) and the major metabolites malate, alanine, and glutamate was similar between mouse and human islets (Figures 1C–1E). However, a small but significant increase in m + 2/m + 3 aspartate and fumarate was detected in mouse islets (Figures 1F and 1G), reflecting an increased contribution of glucose-derived pyruvate into the TCA cycle via acetyl-coenzyme A (CoA). Total aspartate and alanine levels did not differ between the species (Figures 1H and 1I), whereas malate and fumarate levels were lower in mouse (Figures 1J and 1K). Glutamate levels were ~3-fold higher in mouse versus human islets, despite similar MIDs, implying that there is a larger contribution of non-labeled glutamate to the total glutamate pool in this species, e.g., through glutamine transport (Figure 1L).

### Lactate generation is higher in human compared to mouse islets

To obtain a higher-definition view of pyruvate management, its contribution to alanine and lactate production was assessed. In both species, glucose incorporation could be detected in m + 2 and m + 3 lactate, derived from the TCA cycle and direct pyruvate conversion, respectively (Figures 1M–1O). While the MID for alanine was similar in islets from both species (Figure 1D), the accumulation of m + 2 and m + 3 lactate was significantly (~6-fold) higher in humans (Figures 1M and 1N). Total lactate was also higher in human than in mouse islets (Figure 1P). Supporting the notion of increased lactate production through glycolytic input in human islets, the m + 3 G-3-P/m + 3 lactate labeling ratio was higher in mouse versus human islets (Figure S1B).

Suggesting that m + 3 lactate is derived mainly from pyruvate, rather than multiple rounds of the TCA cycle, were the following findings: (1) m + 4 malate and m + 4 aspartate were significantly lower compared to m + 2 and m + 3 isotopomers (Figures 1C and 1F); and (2) m + 4 malate and m + 4 aspartate were similar in human and mouse, despite higher m + 3 lactate in human (Figures 1C–1F and 1M). In addition, previous tracing studies have shown m + 3 lactate accumulation in iPSC-derived islets and human islets after 1 h of tracing.^28^

### High-resolution annotation of ^13^C_6_ glucose-tracing data

**Figure 2.**
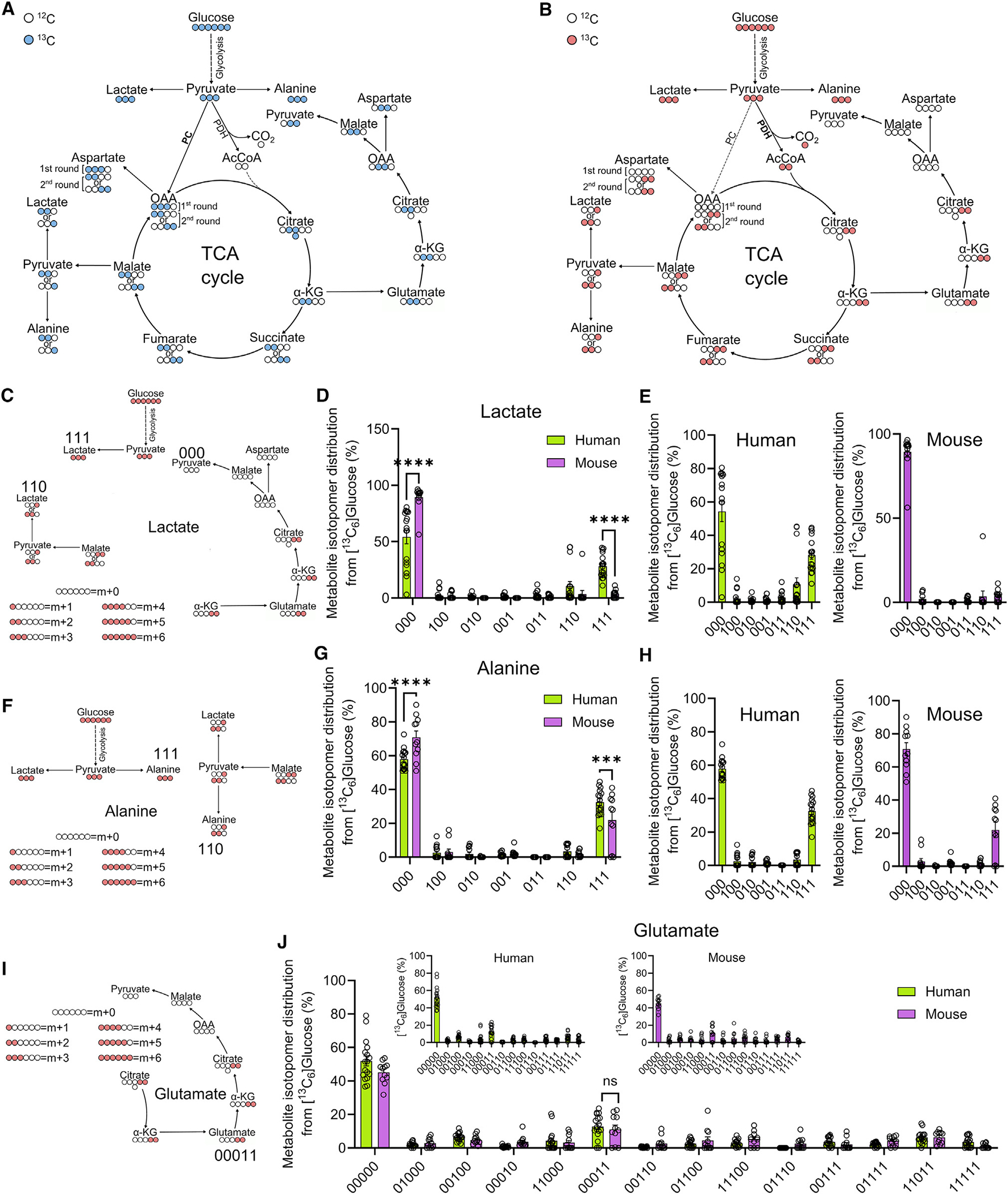
Incorporation of ^13^C from ^13^C_6_ glucose into TCA cycle metabolites through PDH and PC (A) White and blue circles, respectively, show the incorporation of ^12^C and ^13^C into TCA cycle metabolites arising from metabolism of pyruvate by PC. (B) White and red circles, respectively, represent ^12^C and ^13^C atoms as incorporated from ^13^C_6_ glucose into the TCA cycle through the conversion of pyruvate to acetyl-CoA by PDH. (C–E) Lactate_000_, lactate_111_, and lactate_110_ are the most abundant isotopomers (C) in both humans and mice (D), although the incorporation of ^13^C from ^13^C_6_ glucose into lactate_111_ is significantly higher in human than mouse islets (D and E) (E shows same data as D, but as separate graphs for clarity). (F–H) ^13^C incorporation into alanine isotopomers (F) is similar in human and mouse islets (G), with alanine_111_ being the most represented labeled isotopomer (G and H) (H shows same data as G, but as separate graphs for clarity). (I and J) The distribution of labeling patterns for glutamate (I) are similar in human and mouse islets (J), with glutamate_00011_ being the most abundant labeled isotopomer in both species (J) (inset shows same data as in J, but as separate graphs for human and mouse). For all data, *n* = 16–17 islet preparations, nine human donors, and *n* = 12–15 islet preparations, seven or eight animals. Data were analyzed using two-way ANOVA and Sidak’s *post hoc* test. Bar graphs show individual datapoints and mean ± SEM. ns, non-significant; ****p < 0.0001.

To identify isotopomer patterns with high resolution, the MID analysis of ^13^C_6_ glucose-traced human and mouse islets was annotated with 2D ^1^H-^13^C HSQC NMR multiplet analysis (Figures 2A and 2B). Labeling patterns are formed within the chemical structure of each metabolite that are specific to the pathway from which they are produced (Figures 2A and 2B). To define the different isotopomer patterns, a numerical notation was used, where the numbers 0 and 1 indicate ^12^C and ^13^C atoms, respectively. Confirming the accuracy of the approach and the robustness of our findings, the accumulation of lactate_111_ (i.e., fully labeled lactate) was significantly higher in human compared to mouse islets, in line with the MID glucose-tracing data (Figures 1M–1O) (Figures 2C–2E).

### TCA cycle fueling depends more on flux through PDH than PC in human and mouse islets

In both human and mouse islets, lactate_110_ made a greater contribution to the m + 2 isotopologue pool than the other isotopomers (Figures 2C–2E). This finding suggests that TCA-derived lactate is produced primarily from the oxidative TCA cycle rather than the reductive metabolism of PC-derived glutamate, from which pyruvate_011_ and then lactate_011_ would arise (Figures 2C–2E). The majority of alanine was either 000 or 111, with only a very minor contribution to the other isotopomers (Figures 2F–2H). As such, the labeled portion of alanine is exclusively produced from pyruvate upstream of the TCA cycle, meaning that the accumulation of pyruvate_110_ from malate_1100_ (i.e., TCA derived) is employed to regenerate cytoplasmic lactate_110_ (Figures 2C–2E and 2F–2H). Alanine_111_ accumulation was ~20% higher in human than mouse islets, reflecting a greater contribution of transamination toward amino acid production (Figures 2F–2H). Supporting the lactate isotopomer data, the contribution of ^13^C_6_ glucose to the labeling patterns of glutamate was found to be similar in humans and mice (Figures 2I and 2J). In both species, the most abundant labeled isotopomer was glutamate_00011_ (Figures 2I and 2J), which is derived from TCA cycle flux through PDH activity.

### LDH is expressed at higher levels in human compared to mouse β cells

To investigate whether LDH protein is expressed in β cells, we performed immunohistochemistry using an antibody with LDHA, LDHB, and LDHC (i.e., total LDH) cross-reactivity. Cytoplasmic LDH staining was detected in human endocrine and exocrine pancreas (Figure S2A). LDH expression was lower in mouse compared to human islets, with slightly higher levels in the exocrine versus endocrine compartments (Figures S2A and S2C). Following 8 weeks of high-fat diet (HFD) feeding, LDH protein expression increased ~2-fold versus age-matched standard diet controls (Figures S2B and S2C). LDH expression also increased in the exocrine compartment during HFD (Figures S2B and S2D). Occasional intra-islet cells were found to express very high LDH levels in both human and mouse, likely representing endothelial cells known to be enriched for LDHA^29^ (Figures S2A and S2B). β cell de-differentiation was confirmed in the same samples using PDX1 immunostaining (Figures S2E and S2F).

Strong LDH staining could be detected in human liver sections derived from patients with metabolic-dysfunction-associated steatohepatitis (Figure S2G). As expected from studies of enzyme activity, LDH expression was higher in the liver than in β cells (Figures S3A–S3C).^8^ All results were confirmed with multiple immunostaining runs, using both 40× and 60× objectives (Figures S2A–S2D and S3A–S3E).

### *LDHB* is expressed in human β cells within the endocrine compartment

**Figure 3.**
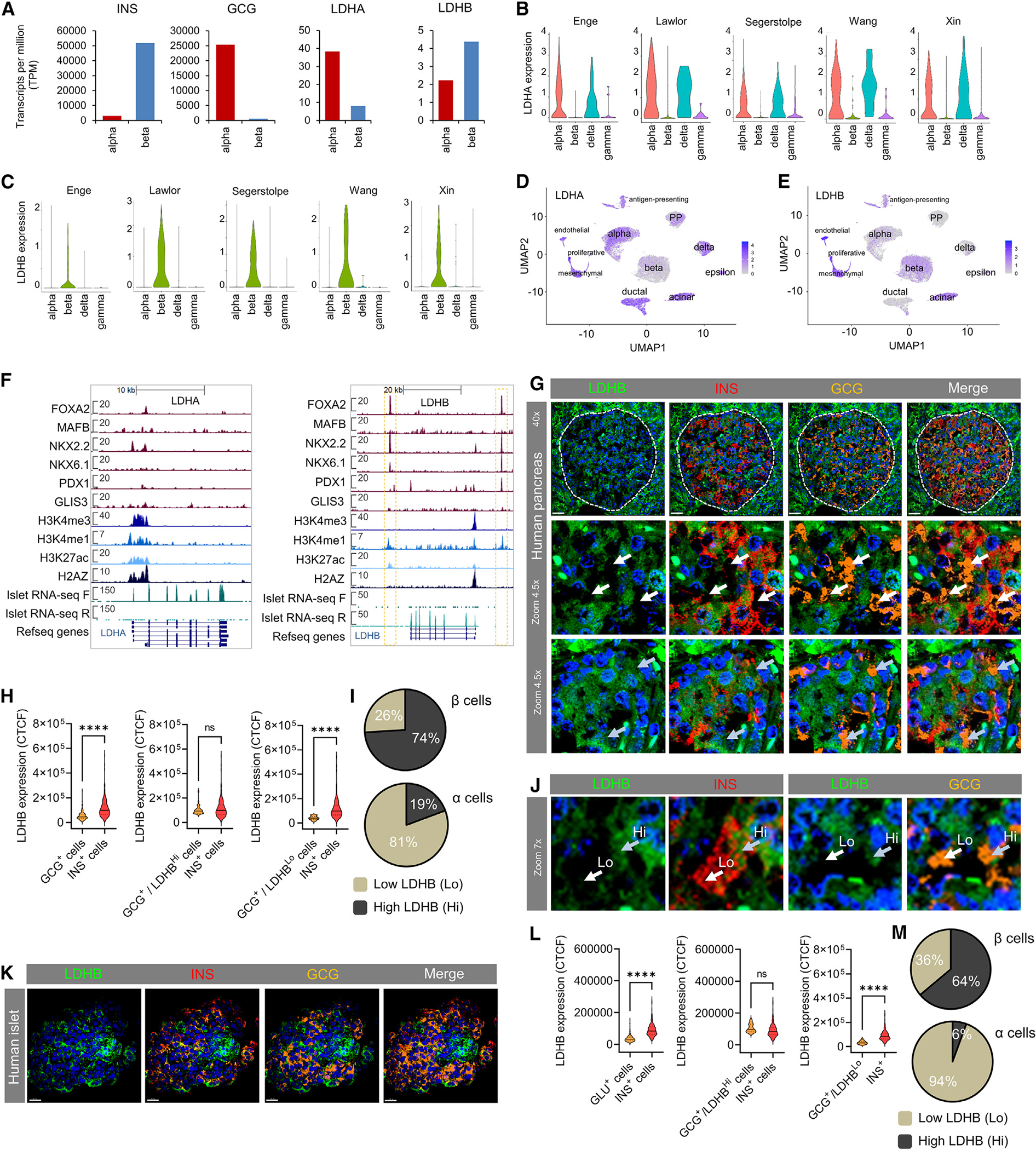
Human β cells specifically express *LDHB* (A) Normalized mRNA levels (transcripts per million [TPM]) for *INS*, *GCG*, *LDHA*, and *LDHB* genes in a and β cells (re-analysis of data from Arda et al.^33^). (B and C) Normalized *LDHA* (B) and *LDHB* (C) expression in α, β, δ, and γ cells from human islet scRNA-seq experiments (*n* = 5 datasets).^29,34–37^ (D and E) Uniform manifold approximation projection (UMAP) plots showing *LDHA* (D) and *LDHB* (E) raw counts clustered according to cell type (*n* = 18 donors).^30,38,39^ (F) Genome browser snapshot of transcription factor binding, histone modification (chromatin immunoprecipitation sequencing [ChIP-seq], targets as indicated), and RNA-seq for *LDHA* and *LDHB*.^40^ (G) LDHB protein expression strongly co-localizes with insulin (INS) expression in human pancreas sections. Zoom-in (middle panel) shows absence of LDHB in glucagon (GCG)+ cells. Zoom-in (bottom panel) shows a small subpopulation of GCG+ cells with detectable LDHB staining. (H) Quantification of LDHB expression in GCG+ and INS+ cells in human pancreas sections, including sub-analysis of GCG+ segregated by high (Hi) and low (Lo) LDHB levels (>8 × 10^4^ CTCF) (*n* = 150 cells, three donors) (Mann-Whitney test). (I) Pie charts showing the proportions of GCG+ and INS+ cells that express Hi or Lo LDHB in human pancreas sections (*n* = 150 cells, three donors). (J) Representative images (zoom in from G) showing LDHB Hi and Lo cells for GCG and INS. (K) As for (G), but showing that LDHB protein expression remains co-localized with INS in isolated human islets. (L and M) As for (H) and (I), but in isolated human islets (*n* = 180 cells, three donors) (Mann-Whitney test). Scale bars, 30 μm. Violin plots show median and interquartile range. Scales in (B) and (C) represent reads per kilobase per million mapped reads (RPKM). ns, non-significant; ****p < 0.0001.

*LDHB* was found to be specifically and highly expressed in β cells (shown also in van Gurp et al.^30^) within the islet, whereas *LDHA* was specifically and highly expressed in α cells (Figure 3A). Analysis of single-cell RNA sequencing (scRNA-seq) data confirmed these findings (Figures 3B–3E). Confirming the accuracy of the clustering-based analysis, the lactate transporter MCT1, encoded by *SLC16A1*, could not be detected in any islet endocrine cell type (Figure S3F), as previously reported.^31,32^

Recent studies have shown that LDHB can compensate for LDHA activity when *LDHA* expression levels are low.^41,42^ However, we cannot exclude the possibility that LDHA may also catalyze pyruvate to lactate conversion in β cells, as they do contain detectable *LDHA* mRNA, based on both scRNA-seq and bulk RNA-seq of α cells and β cells (Figures 3A–3E). This is consistent with the open chromatin conformation and transcription factor binding to the *LDHA* promoter in the human islet (Figure 3F). By contrast, *LDHB* possessed two specific enhancers within the CCCTC-binding factor boundaries, suggestive of β cell-specific regulation (Figure 3F).

### LDHB protein localizes predominantly to human β cells within the endocrine compartment

Immunohistochemistry was performed using an antibody against LDHB, validated by the human tissue atlas using tandem mass tag mass spectrometry, protein array, and subcellular localization.

Similarly to total LDH (i.e., LDHA + LDHB + LDHC), LDHB was located throughout the cytoplasm (Figure 3G). In line with the transcriptomic analysis, LDHB protein could be readily observed throughout the β cell compartment but was undetectable in the majority (81%) of α cells (Figures 3G–3I). We did, however, notice a small subpopulation (~19%) of α cells with high levels of LDHB (Figures 3G–3J). Likewise, a proportion of β cells could be differentiated by their absent or low expression of LDHB (26%) (Figures 3G–3J). All results were confirmed with multiple immunostaining runs, using both 40× and 60× objectives (Figures 3G–3J, S3G, and S3H). Almost identical results were obtained in isolated human islets, suggesting that isolation and culture time do not influence LDH/LDHB and *ergo* lactate levels (Figures 3K–3M and S3I–S3K).

Confirming antibody specificity, a 3-fold reduction in LDHB expression could be seen in EndoC-βH1 cells treated with small interfering RNA against *LDHB* versus control (Figures S4A and S4B).

### LDHB limits LDHA-dependent lactate generation in human β cells

**Figure 4.**
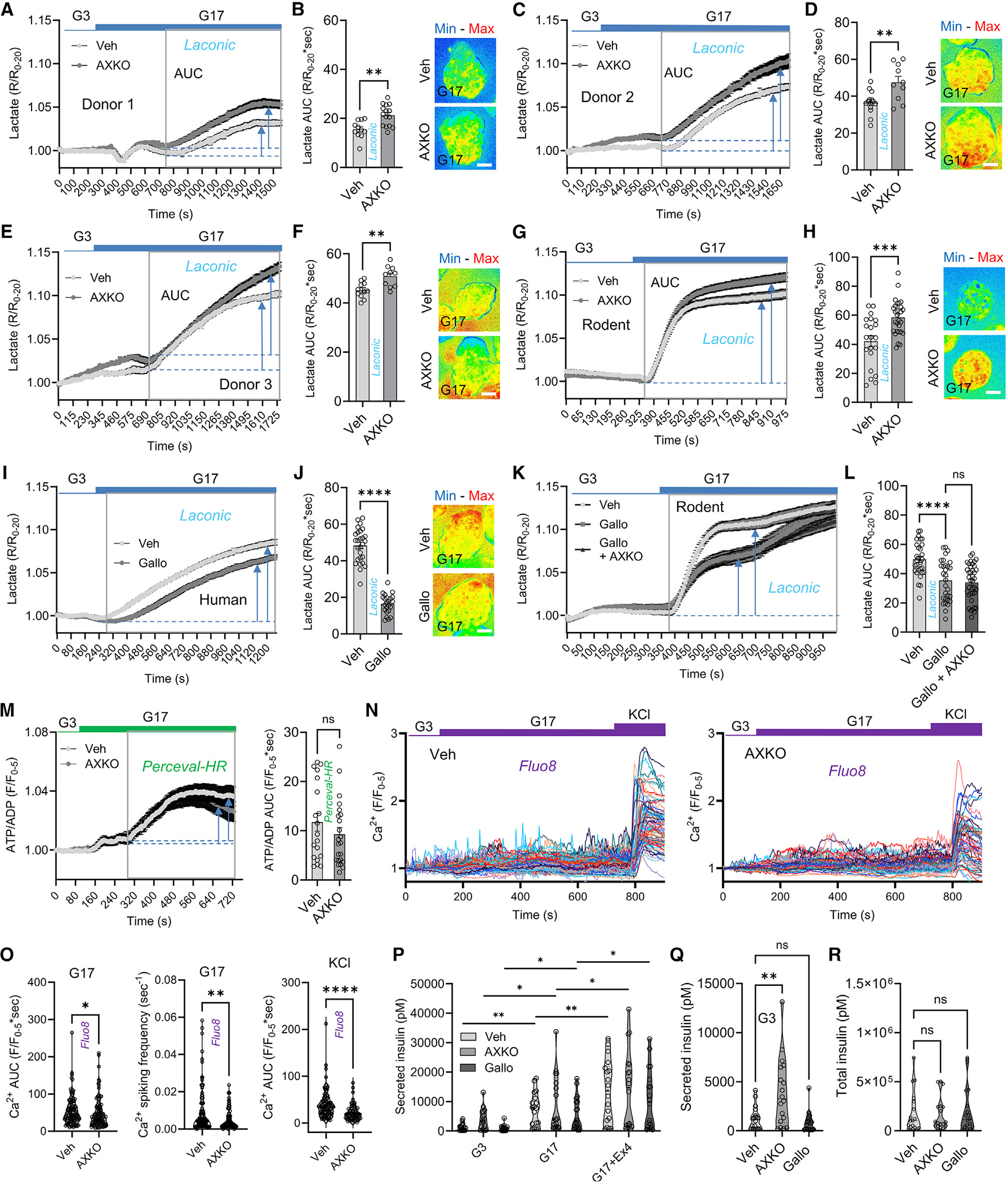
Effects of LDHB inhibition on lactate levels and function in human β cells (A–F) Traces (A, C, and E) and bar graph and representative images (B, D, and F) showing that glucose-stimulated lactate generation is amplified by 10 μM AXKO-0046 (LDHB inhibitor) in islets from three separate donors (donor 1, *n* = 10–15 islets; donor 2, *n* = 10–14 islets; donor 3, *n* = 10–12 islets) (unpaired t test). (G and H) AXKO-0046 amplifies glucose-stimulated lactate generation in mouse β cells, as shown by mean traces (G) and summary bar graph and representative image (H) (*n* = 21–28 islets, three animals) (unpaired t test). (I and J) 10 μM galloflavin (LDHA + LDHB inhibitor) decreases glucose-stimulated lactate generation in human β cells, as shown by mean traces (I) and summary bar graph and representative image (J) (*n* = 23–27 islets, three donors) (unpaired t test). (K and L) AXKO-0046 does not increase glucose-stimulated lactate generation in the presence of galloflavin in mouse β cells, as shown by mean traces (K) and summary bar graph (L) (*n* = 29–38 islets, three animals) (one-way ANOVA, Sidak’s *post hoc* test). (M) AXKO-0046 does not significantly influence glucose-stimulated ATP/ADP ratios in human β cells (*n* = 19–23 islets, three donors) (unpaired t test). (N and O) Traces (N) and violin plots (O) showing that AXKO-0046 has a small but significant effect on glucose- and KCl-stimulated Ca^2+^ levels in human β cells (*n* = 101–118 cells, three donors) (Mann-Whitney test). (P) AXKO-0046 and galloflavin do not significantly influence glucose- or Ex4-stimulated insulin secretion (*n* = 18 repeats, three donors) (two-way repeated measures ANOVA, Tukey’s *post hoc* test). (Q) Same data as in (P) G3 but, for the sake of clarity, separate analysis showing that AXKO-0046, and not galloflavin, significantly increases basal insulin secretion (one-way ANOVA, Sidak’s *post hoc* test). (R) Insulin content is similar between all states examined (Kruskal-Wallis test, Dunn’s *post hoc* test). Traces show mean ± SEM. Bar graphs show individual datapoints and mean ± SEM. Violin plots show individual datapoints and median. Scale bars, 83 μm. Arrows and box show AUC calculation boundaries. ns, non-significant; *p < 0.05; **p < 0.01; ***p < 0.001; ****p < 0.0001. Veh, vehicle; AXKO, AXKO-0046; Gallo, galloflavin, G3, 3 mM glucose; G17, 17 mM glucose; Ex4, exendin4.

Human islets transduced with a β cell-specific lactate fluorescence resonance energy transfer (FRET) sensor^43,44^ responded to 17 mM glucose with an increase in intracellular lactate levels (Figures 4A–4F). Islets were pre-incubated for 2 h in vehicle or 10 μM AXKO-0046, a specific LDHB inhibitor with no detectable LDHA activity.^45^ A small (10%–20%) but replicable increase in glucose-stimulated lactate generation was observed in AXKO-0046-treated islets (Figures 4A–4F). Similar results were observed in mouse β cells, which express LDHB, albeit at lower levels than human β cells (Figures 4G and 4H). By contrast, pre-incubation with 10 μM galloflavin, an LDHA + LDHB inhibitor,^46^ impaired glucose-stimulated lactate generation in human β cells (Figures 4I and 4J).

Demonstrating specificity of LDHB inhibition, AXKO-0046 was unable to influence lactate levels in mouse islets pre-treated with galloflavin (Figures 4K and 4L). Furthermore, AXKO-0046 and galloflavin did not influence apoptosis versus vehicle, suggesting that LDHA and/or LDHB are dispensable for cell survival (Figures S4C and S4D).

Together, these data demonstrate that LDHA is the major driver of glucose-stimulated lactate generation in human β cells, and that LDHB limits this effect to maintain lactate within a tight range.

### LDHB inhibition influences Ca^2+^ fluxes but not ATP/ADP ratios

We next looked at whether LDHB inhibition influences glucose-stimulated ATP/ADP and Ca^2+^ rises in human β cells. AXKO-0046 was unable to significantly influence glucose-stimulated ATP/ADP ratios in human islets (Figure 4M). By contrast, both glucose- and KCl-stimulated Ca^2+^ fluxes were blunted in AXKO-0046-versus vehicle-treated islets (Figures 4N and 4O). Detailed analysis of glucose-stimulated Ca^2+^ oscillations showed that AXKO-0046 reduced both area under the curve (AUC) and spiking frequency (Figures 4N and 4O), in keeping with the K_ATP_ channel-opening effects of lactate.^13^

We attempted to replicate experiments in LDHB-expressing EndoC-βH5 spheroids (Figure S4E). EndoC-βH5 spheroids, however, showed signs of blebbing following pre-incubation with 10 μM AXKO-0046. Even at 100 nM, AXKO-0046 suppressed both glucose-stimulated Ca^2+^ fluxes and ATP/ADP ratios (Figures S4F–S4I). Transcriptomic data revealed that EndoC-βH5 spheroids express moderate LDHA levels, which might explain the exaggerated effects of LDHB inhibition versus primary human β cells (64.11 ± 2.60 versus 247.73 ± 5.00 TPM, *LDHA* versus *LDHB*; mean ± SD; taken from GEO: GSE224732)^47^. Thus, high levels of LDHB are needed in human β cells to limit LDHA-induced lactate generation, which would otherwise be metabolically destructive.

### LDHB prevents inappropriate basal insulin secretion

To understand the contribution of LDHB and lactate to insulin release in human islets, secretion assays were performed following treatment with vehicle, AXKO-0046, or galloflavin. Neither AXKO-0046 nor galloflavin significantly influenced glucose-stimulated or exendin4-stimulated insulin secretion (Figure 4P). We noticed, however, that basal insulin secretion was much higher in samples treated with AXKO-0046 (Figures 4P and 4Q). Supporting a predominant role for LDHB versus LDHA in regulating human β cell function, galloflavin did not affect basal insulin secretion (Figures 4P and 4Q). Total insulin content was similar between all conditions (Figure 4R).

### LDHB expression is associated with elevated fasting insulin in human

To provide human genetic evidence for a role of LDHB in β cell function, we performed Mendelian randomization analyses of *LDHB cis*-EQTLs for type 2 diabetes, glucose, and insulin secretion genome-wide association study (GWAS) outcomes. While *LDHB cis*-EQTLs did not associate with type 2 diabetes, 2-h glucose, or fasting glucose, there was a strong association with fasting insulin and hemoglobin A1c (HbA1c) (Table S1A–C). Notably, *LHDB cis*-EQTLs for decreased *LDHB* expression—i.e., aligned to our LDHB-inhibition experiments—were associated with increased fasting insulin (Tables S1A–S1C).

## DISCUSSION

The observation that the human islet lactate pool is derived from both TCA cycle- and pyruvate-derived sources suggests that mechanisms must be in place for pyruvate conversion. In many tissues, pyruvate would be converted to lactate by LDH; however, lactate generation is minimal in purified rat β cells,^48^ and the *Ldha* isozyme of the enzyme has been shown to be disallowed or absent in the murine pancreatic β cell.^8,10,49^ In keeping with these findings and further supporting a more limited role for LDH in rodents, lactate and LDH protein could only be detected at low levels in mouse islets, although glucose-stimulated lactate rises could still be detected by us and others in single β cells.^44^ By contrast, human β cells were found to specifically and strongly express *LDHB*/LDHB, confirming previous single-cell screening studies by van Gurp et al.^30^ *LDHA* expression was also detected at low-moderate levels based upon bulk RNA-seq of purified human β cells.

It is possible that α cells contribute to the accumulation of lactate. Human α cells account for ~35% of the islet and express *LDHA* at levels six times higher than β cells.^50,51^ However, a major source of α cell lactate is via monocarboxylate transporters,^9,52,53^ which are unlikely to play a role here as lactate was absent from the tracing medium. In addition, while the total amount of lactate was only doubled in humans compared to mice, the m + 2 (TCA-derived) and m + 3 (glycolytically derived) lactate accumulation was ~6-fold higher in human versus mouse islets, which cannot be accounted for solely by differences in α cell proportion. Lastly, studies with a lactate FRET sensor showed that glucose-stimulated human β cells are capable of generating significant intracellular lactate levels. Taken together, these data suggest that α cell lactate only makes a minor contribution to the whole m + 3 and m + 2 lactate increase detected here. We cannot exclude a contribution of the exocrine and other compartments to the findings here, since human islet preps also typically comprise acinar, duct, and other non-islet cells, where *LDHA/LDHB* is enriched versus islet cells (see Figures 3D and 3E).

Human and mouse islets display a greater accumulation of lactate_110_, rather than lactate_011_. While the accumulation of lactate_110_ is indistinguishable in the PDH- and PC-mediated TCA cycle, the 011 isotopomer would only derive from the reductive metabolism of PC-derived glutamate. Corroborating this, the major glutamate isotopomer derived from exogenous ^13^C_6_ glucose was glutamate_00011_. Although glutamate is not a TCA cycle metabolite, it is in rapid exchange with a-KG and can be used as a readout of TCA cycle flux through PDH or PC. Consequently, the accumulation of glutamate_00011_ provides further evidence for a higher reliance of the TCA cycle on the activity of PDH, rather than PC. Although PC and PDH were thought to contribute equally to the TCA cycle in β cells,^20–22^ previous studies have shown that high glucose concentrations *in vitro*, more reflective of those seen post-prandially, are associated with an increase toward PDH activity.^11,15,54^ Our studies thus show that the relative contribution of PC to the TCA cycle is lower than PDH (~20%), confirming previous findings.^11,15^ This does not mean that PC flux is unimportant, since PC is much more responsive to glucose stimulation than PDH.^11^ While anaplerosis through PC is relatively limited in the β cell, we note that glucose carbons can repeatedly transit the PEP cycle to increase ATP/ADP in the cytosol.^11^ As such, PC makes disproportionate contributions to K_ATP_ channel closure, and hence the triggering phase of insulin secretion, by generating plasma-membrane-localized increases in ATP/ADP.^14^ Moreover, PC might make more important contributions to anaplerosis in stressed human β cells in which increased PC activity reduces NO production to counteract inflammation.^55^ PC is also important for funneling pyruvate into the generation of the glucose-stimulated insulin secretion (GSIS)-coupling factors citrate and isocitrate, which sustain 2-KG and NADPH production.^18^ Nonetheless, insulin secretion is not the only energy sink on the β cell, and glucose flux through PDH is likely to provide a source of acetyl-CoA to support other demands, such as ion pumping and protein synthesis.^48,56^

What might be the role of direct pyruvate to lactate conversion in pancreatic β cells? Our data clearly show that lactate levels have a bearing on glucose- and KCl-stimulated Ca^2+^ oscillations and basal insulin secretion. In addition, LDHB activity might also provide a source of reducing equivalents to support other NADH-producing metabolic pathways. Providing evidence for an important contribution of pyruvate to lactate conversion in NADH/NAD+ balance, the most abundant lactate isotopomers were lactate_111_ and lactate_110_, which respectively represent lactate generation upstream and downstream of the TCA cycle. Since the conversion of pyruvate to lactate is associated with the generation of cytosolic NAD^+^, higher levels of total lactate_111_ in humans might reflect an increase in the activity of NADH-producing pathways relative to rodents. Notably, the alanine isotopomer distribution showed accumulation of alanine_000_ and alanine_111_, suggesting that pyruvate accumulated downstream of the TCA cycle is employed in the regeneration of lactate_110_. Arguing against this possibility, β cells are unable to extracellularly transport lactate and already have a large capacity to produce reducing equivalents, e.g., via the glycerol phosphate and malate-aspartate shuttle.^57^ Although membrane-associated LDH was found to form nanodomains with K_ATP_ channels and support the local production of NAD^+^ for GAPDH, the contribution of LDH activity (at least in mouse β cells) was small relative to other unidentified plasma-membrane-associated NADH oxidases that support this function. Lastly, significant pools of cytosolic pyruvate and lactate, and sufficient LDH to equilibrate these pools, might provide a buffer to minimize wide fluctuations in NADH/NAD (or NAD(P)H/NADP), offering some protection against oxidative stress.^58^

In summary, we show that LDHB acts to limit human β cell lactate levels, prevent inappropriate insulin release at low glucose concentration, and shape glucose-stimulated Ca^2+^ fluxes.

### Limitations of the study

There are a number of limitations in the present studies. Firstly, glucose-tracing studies in purified α cells and β cells are warranted, although they should be interpreted in light of loss of cell-cell interactions and changes in cell phenotype. Secondly, glucotoxicity might induce the upregulation of disallowed genes in the β cell.^59^ However, *LDHA* or *LDHB* were not differentially expressed in human islets chronically exposed to 22.2 mM glucose.^60^ Thirdly, glucose tracing should be performed at different time points, similarly to recent studies.^27,28^ Fourthly, functional studies depended on small-molecule chemical inhibitors, and should be repeated in primary human β cells silenced for LDHA/LDHB.

## STAR★METHODS

Detailed methods are provided in the online version of this paper and include the following:

### RESOURCE AVAILABILITY

#### Lead contact

Further information and requests for resources and reagents should be directed to and will be fulfilled by the lead contact, David J. Hodson (david.hodson@ocdem.ox.ac.uk).

#### Materials availability

This study did not generate any new unique reagents.

#### Data and code availability

- This paper analyses existing, publicly available data. Accession numbers for the datasets are listed in the key resources table.
- This paper does not report original code.
- Any additional information required to reanalyze the data reported in this paper is available from the lead contact upon request.

### EXPERIMENTAL MODEL AND STUDY PARTICIPANT DETAILS

#### Mice

Male 8- to 12-week-old CD1 mice (Charles River stock no. 022) were used as tissue donors. Mice were socially-housed in specific-pathogen free conditions under a 12 h light-dark cycle with *ad libitum* access to food and water, relative humidity 55 ± 10% and temperature 21 ± 2°C.

Animal studies were regulated by the Animals (Scientific Procedures) Act 1986 of the U.K. (Personal Project Licences P2ABC3A83 and PP1778740). Approval was granted by the University of Birmingham and University of Oxford Animal Welfare and Ethical Review Bodies (AWERB).

#### Human

Human islets (Lille): human pancreatic tissues were harvested from brain-dead adult donors in accordance with the Lille clinical islet transplantation program’s traceability requirements (clinicaltrials.gov, NCT01123187, NCT00446264, NCT01148680), and were approved in agreement with French regulations and the Ethical Committees of the University of Lille and the Center Hospitalier Régional Universitaire de Lille.

Human islets (Milan): the use of human islets for research was approved by the Ethics Committee of San Raffaele Hospital in Milan (IPF002-2014).

Human islets (Oxford): human pancreata were retrieved from Donors after Brain-Stem Death (DBD) with appropriate consent and ethical approval under 09/H0605/2 (REC: Oxfordshire Rec B). Islets were isolated in the DRWF Oxford Human Islet Isolation Facility using established isolation methods.

Human islets (Alberta^65^): islet isolation was approved by the Human Research Ethics Board at the University of Alberta (Pro00013094). All donors’ families gave informed consent for the use of pancreatic tissue in research.

Human pancreas sections (Oxford): postmortem pancreas samples were obtained from donors, with appropriate permissions registered under CUREC R83564/RE001.

Studies with human islets and pancreata were approved by the University of Birmingham Ethics Committee, the University of Oxford Ethics Committee, as well as the National Research Ethics Committee (REC 16/NE/0107, Newcastle and North Tyneside, UK).

Liver sections (Oxford): Authorization for research use of steatotic livers unsuitable for transplantation was obtained by a specialist nurse in organ donation in accordance with NHSBT guidelines, with appropriate permissions registered under REC 14/LO/0182. The study was approved by the North East – Tyne and Weir South research ethics committee (16/NE/0248) and by the NHSBT Research, Innovation and Novel Technologies Advisory Group.

### METHOD DETAILS

#### Study design

No data were excluded, and all individual data points are reported in the figures. The measurement unit is animal or donor, with experiments replicated independently on different days. Islet isolation is a nuisance variable and as such data are taken from independent islet preparations. Samples and animals were allocated to treatment groups in a randomized manner to ensure that all states were represented in the different experiment arms. MID analysis was performed by a user blinded to sample identity. For metabolic tracing, nine samples per investigated state are required to correctly reject the null hypothesis (two-tailed test) for an effect size (d) = 1.5 (calculated in GPower 3.1).

#### Mouse islets

Animals were culled using a schedule-1 method followed by injection of the common bile duct with 1 mg/mL collagenase NB 8 (Serva) in RPMI 1640 (Gibco) and pancreas dissection. After dissection, the pancreas was incubated in a water bath at 37°C for 12 min. Subsequently, the tissues were shaken in 15 mL of RPMI 1640 and centrifuged for 1 min at 1500 rpm three times to induce mechanical digestion. Islets were separated using Histopaque 1119 and 1083 (Sigma-Aldrich) gradients, before hand-picking and culture. Unless otherwise stated, the islets obtained were kept in culture in RPMI 1640 supplemented with 10% fetal bovine serum (FBS, Gibco), 100 units/mL penicillin, and 100 μg/mL streptomycin (Sigma-Aldrich), at 37°C and 5% CO_2_.

For immunohistochemistry, sections were cut from formalin-fixed, paraffin-embedded (FFPE) pancreata obtained from wild-type male and female C57BL6 mice fed standard diet or high fat diet (60% fat, Research Diets) for 8–12 weeks. To reduce the number of animals used in experiments in line with NC3Rs policy, blocks were re-used from a previous study,^66^ new sections cut at different depth, and immunostained with different markers (LDH, PDX1, see below).

#### EndoC-βH1 and EndoC-βH5 cells

Total protein was extracted from EndoC-βH1 cells using RIPA lysis buffer. Lysis buffer was supplemented with EDTA-free protease inhibitor and phosphatase inhibitor cocktails. Lysates were resolved on SDS–PAGE, transferred to a nitrocellulose membrane using the Trans-Blot Turbo Transfer Pack, blocked in 5% milk for 1 h, followed by incubation with a polyclonal anti-LDHB antibody (HPA019007) at 4°C overnight. After washing, membranes were incubated for 2 h at room temperature with secondary antibody conjugated to horseradish peroxidase (HRP). After incubation with Clarity Western ECL Substrate, bands were detected with a BioRad GelDoc Biosystems Western Blotting imager.

EndoC-βH1 cells were transfected using Lipofectamine RNAiMAX with ON-TARGETplus siRNA targeting *LDHB* (L-009779-00, Dharmacon, Horizon Discovery) at a final concentration of 80 nM. Non-targeting control pool (D-001810-10, Dharmacon, Horizon Discovery) was used as a control. Briefly, siRNA and Lipofectamine RNAiMAX were combined in OptiMEM and applied to the cells. Medium was changed 2.5 h later for fresh EndoC-βH1 culture medium. Cells were harvested in RIPA lysis buffer for western blotting, or in RLT lysis buffer for mRNA purification (Qiagen). Western blotting for LDHB was performed as described above.

Primers used for PCR were as follows: PPIA (housekeeping) Fw primer: ATGGCAAATGCTGGACCCAACA, PPIA (housekeeping) Rv primer: ACATGCTTGCCATCCAACCACT, LDHB Fw primer: TGATGGATCTGCAGCATGGG and LDHB Rv primer: CAGATTGAGCCGACTCTCCC.

To generate EndoC-βH5 (Human Cell Design) pseudoislets, 1 million cells were seeded in Aggrewell Multiwell plates in ULT1β1 culture media (Human Cell Design) at 37°C and 5% CO2. Pseudoislets aggregated in 3–4 days and media was changed every 2 days.

#### Human islets

Islets were cleared of possible debris via filtration with a 40 μm cut-off filter, hand-picked and cultured in CMRL medium (Corning) containing 5.5 mM glucose, 10% FBS, 100 units/mL penicillin, 100 μg/mL streptomycin and 0.1% amphotericin B (Sigma-Aldrich) at 37°C and 5% CO_2_. Human donor characteristics are reported in Table S2.

#### ^13^C_6_ glucose tracing

For ^13^C_6_ glucose tracing, 60 (for GC-MS) or 150–230 (for NMR) islets were used. Isolated islets were cultured in RPMI 1640, no glucose medium (Gibco), supplemented with 10% BSA, 10% FBS, 100 units/mL penicillin, and 100 μg/mL streptomycin plus 10 mM ^13^C_6_ glucose (Sigma-Aldrich). After 24 h, the metabolites were extracted adding HPLC-grade methanol, HPLC-grade distilled H_2_O containing 1 μg/mL D6-glutaric acid and HPLC-grade chloroform (all from Sigma-Aldrich) in a 1:1:1 ratio, to the islets. Following centrifugation, the polar fractions were collected and vacuum dried before either GC-MS or NMR analyses. A 24 h tracing duration was used to allow steady state labeling of both glycolytic and TCA metabolites in the same sample, as well as sufficient ^13^C incorporation for NMR-based labelling pattern annotation. While antibiotics have been shown to influence mitochondrial function, research islet isolation is a non-sterile procedure. Antibiotic use is therefore justified, since low grade infection is likely to exert a larger (and unnoticed) effect on mitochondrial activity.

#### GC-MS

The dried polar extracts were prepared for GC-MS analysis through solubilization in 40 μL of 2% methoxyamine hydrochloric acid in pyridine (Fisher Scientific) at 60°C for 60 min and derivatization with 60 μL of N-tertbutyldimethylsilyl-N-methyltrifluoroacetamide (MTBSTFA) with 1% tertbutyldimethyl-chlorosilane (TBDMCS) (both from Sigma-Aldrich). The suspension was further incubated at 60°C for 60 min, before being centrifuged at 13300 rpm for 10 min at 4°C and transferred to chromatography vials with a glass insert (Restek) for GC-MS analysis. The samples were analyzed on an Agilent 8890 GC and 5977B MSD system. To do this, 1 μL of sample was injected in splitless mode with helium carrier gas at a rate of 1.0 mL/min. The compound detection was carried out in scan mode and total ion counts of each metabolite were normalized to the internal standard D6-glutaric acid and corrected for natural ^13^C abundance.^67^ Retention times, ion counts and MID data are provided in Tables S3A–S3C (mouse) and Tables S4A–S4C (human).

#### NMR spectroscopy

Following the ^13^C_6_ glucose tracing, the dried polar metabolites were resuspended in 60 μL of phosphate buffer: 57.8 mM disodium phosphate (Na_2_HPO_4_, Sigma-Aldrich), 42.2 mM monosodium phosphate (NaH_2_PO_4_, Sigma-Aldrich), 0.5 mM 3-(trimethylsilyl) propionic-2,2,3,3-d4 acid sodium salt (D4-TMSP, Sigma-Aldrich) in deuterium oxide (D_2_O, Sigma-Aldrich). Subsequently, the samples were centrifuged for 10 min at 14800 rpm and sonicated in an ultrasonic bath for 5 min, before being loaded into NMR tubes (outer diameter: 1.7 mm, Bruker) for acquisition. A Bruker *Neo* 800 MHz NMR spectrometer equipped with a 1.7 mm z-PFG TCI Cryoprobe was used to acquire 2D ^1^H,^13^C-HSQC NMR spectra. The HSQC spectra were acquired with echo/anti-echo gradient coherence selection with an additional pre-saturation for suppressing the residual water resonance. The spectral widths were 15.6298 ppm and 189.7832 ppm in the ^1^H and ^13^C dimension, 512 complex data points were acquired for the ^1^H dimension and 25% (512) out of 2048 complex data points were acquired for the ^13^C indirect dimension using a non-uniform sampling scheme. Apparent ^13^C,^13^C J-coupling was enhanced 4-fold. The interscan relaxation delay was set to 1.5 s 2D ^1^H,^13^C-HSQC spectra were reconstructed via the compressed sensing IRLS algorithm using the MddNMR (version 2.5)^62^ and NMRPipe (version 9.2)^63^ software. All NMR spectra were analyzed in the MATLAB based MetaboLab software package.^64^

#### Immunohistochemistry

FFPE pancreas, liver and isolated islets were cut at 5 μm and deparaffinized. Human donor characteristics are reported in Table S5. PBS with 2% BSA and 0.2% Triton X-100 was used to block sections for 1 h at room temperature. Heat induced antigen retrieval was performed with 10 mM citrate buffer (pH6) using a microwave for 10 min and cooled for 10 min in cold water. Sections were incubated with primary antibodies overnight at room temperature, followed by washes with PBS containing 2% BSA and 0.2% Triton X-100. Secondary antibodies were incubated for 2 h at room temperature. Sections were mounted with DAPI (Vectashield, cat no. H-1500) and stored at 4°C. Primary antibodies used were mouse anti-LDH (Santa Cruz Biotechnology, cat no. sc-133123; RRID: AB_2134964), rabbit polyclonal anti-LDHB (Sigma-Aldrich, cat no. HPA019007; RRID:AB_2670008), guinea-pig anti-PDX1 (Abcam, cat no. ab47308; RRID:AB_777178), guinea pig anti-insulin (Abcam, cat no. ab7842; RRID: AB_306130), and mouse monoclonal anti-glucagon (Sigma-Aldrich, cat no. G2654; RRID:AB_259852). Human and mouse LDHA, LDHB and LDHC share 94.0%, 97.9% and 74.5% protein sequence identity, respectively. We note however that LDHC is undetectable in the human pancreas^68^ and is largely confined to the testis.^69^ For LDHA and LDHB detection, we used an amplification step consisting of anti-mouse biotinylated (DAKO, cat no. E0413) or anti-rabbit biotinylated (NOVEX, cat no. A16039; RRID: AB_2534713) antibodies, followed by incubation with streptavidin-FITC (Sigma-Aldrich, Cat no. S-3762). For all other markers, secondary antibodies used were anti-guinea pig Alexa Flour 568 (Thermo Fisher, cat no. A-11075; RRID: AB_141954), anti-guinea pig Alexa Flour 647 (Thermo Fisher, cat no. A-21450; RRID: AB_141882) and anti-mouse Alexa Flour 568 (Thermo Fisher, cat no. A-11004; RRID: AB_2534072).

Sections were imaged using an Olympus FV3000 confocal microscope equipped with high-sensitivity spectral detectors and 40x, 1.25 NA and 60x, 1.30 NA silicone objectives. Excitation and emission wavelengths for DAPI, Alexa Fluor 488, Alexa Flour 568 and Alexa Flour 647 were λex = 405 nm/λem = 406–461 nm, λex = 488 nm/λem = 499–520 nm, λex = 561 nm/λem = 579–603 nm, λex = 640 nm/λem = 649–700 nm respectively.

#### Lactate, Ca^2+^ and ATP/ADP imaging

Isolated islets were transduced 24–48 h with the β cell specific lactate FRET sensor, Ad-RIP-Laconic,^43,44^ prior to widefield imaging using a Nikon Ti-E base equipped with 89 North LDI-7 Laser Diode Illuminator, 25x/0.8 NA objective and Prime BSI Express sCMOS. Excitation was delivered at λ = 445 nm and emission detected at λ = 460–500 and λ = 520–550 nm for mTFP and Venus, respectively. All experiments were performed in HEPES-bicarbonate buffer containing (in mmol/L) 120 NaCl, 4.8 KCl, 24 NaHCO_3_, 0.5 Na_2_HPO_4_, 5 HEPES, 2.5 CaCl_2_, 1.2 MgCl_2_, supplemented with 3–17 mmol/L D-glucose, and bubbled with 95% O_2_/5% CO_2_. Laconic intensity was calculated as the ratio of mTFP/Venus, and normalized as R/R_0–20_ where R = fluorescence intensity at any given timepoint, and R_0–20_ = mean fluorescence intensity between frames 0–20. AXKO-0046 (MedChemExpress, cat no. HY-147216) was used as a selective nanomolar-affinity small molecule inhibitor of LDHB (IC_50_ = 42 nM, IC_MAX_ = 10^−5^ nM).

Ca^2+^ and ATP/ADP imaging was performed in human islets and EndoC-βH5 spheroids loaded with Fluo8 or transduced with Ad-RIP-Perceval-HR, respectively.^11,61^ Islets were imaged as above, with excitation delivered at λ = 470 nm using a CrestOptics X-light V2 spinning disk head; and emission detected at λ = 500–550 nm. Ca^2+^ and ATP/ADP traces were normalized as F/F_0–5,_ F/F_0–20_ or F/F_min_ where F = fluorescence intensity at any given timepoint, and F_0–5_/F_0–20_/F_min_ = mean fluorescence intensity between frames 0–5 or 0–20, or minimum fluorescence, respectively. For all timelapse imaging, frame rate was 0.33 Hz_._

#### TUNEL assay

TUNEL staining was performed using a DeadEnd Fluorometric TUNEL System (Promega), according to the manufacturer instructions. The proportion of apoptotic cells was calculated as the area of TUNEL+ staining (fluorescein-12-dUTP)/islet area. Images were captured using a Leica SP8 confocal microscope and 63× 1.40 NA oil objective. Excitation and emission wavelengths for DAPI and fluorescein-12-dUTP were λex = 405 nm/λem = 410–643 nm, λex = 488 nm/λem = 498–731 nm.

#### Insulin secretion

Batches of 15 human islets were sequentially stimulated with 3 mM glucose, 17 mM glucose or 17 mM glucose +20 nM Exendin-4 according to IsletCore protocols IO (Static Glucose-stimulated Insulin Secretion (GSIS) Protocol - Human Islets V.2). Total insulin was extracted using acid ethanol and insulin concentration determined using Lumit Insulin kit according to the manufacturer’s instructions.

#### Mendelian randomization

*Cis*-instrument Mendelian randomization (MR)^70–72^ was used to assess the impact of pancreatic LDHB expression on type 2 diabetes (T2D) and glycemic traits. The MR analyses used publicly available genome-wide association study (GWAS data) from cohorts comprised of participants of European ancestry, which have existing ethical permissions from their respective internal review boards and include participant informed consent with rigorous quality control.

Expression quantitative trait loci (eQTL) data for bulk pancreatic tissue were obtained from the Genotype-Tissue Expression project (GTEx) version 8 data from donors of European ancestry (N = 243).^73^ The genetic instrument for LDHB expression was constructed with one independent (linkage disequilibrium [LD] R^2^ < 0.1 single nucleotide polymorphisms (SNPs) associated with pancreatic LDHB expression located within ± 100 kilobases) of the *LDHB* locus (chromosome 12:21,788,276–21,910,791). Next, the LDHB SNP was extracted from the outcome datasets, including T2D (80,154 cases/853,816 controls),^74^ glycated hemoglobin (HbA1c) (N = 361,194)^75^ and other recent GWASs of glycemic markers (2-h glucose, fasting glucose, and fasting insulin) from MAGIC (Meta-Analyses of Glucose and Insulin-related traits) Consortium (N ≤ 200,622).^76^

Exposure and outcome data were cleaned and harmonized (i.e., the effect alleles and corresponding directions of the regression coefficient were aligned) and the Wald ratio^77^ was used to calculate the MR estimates. LDHB expression is measured in transcripts per million (TPM)^73^ and MR estimates correspond to an increase in a 1-standard deviation change in circulating glycemic marker or change in risk for T2D per TPM increase in pancreatic LDHB expression. All MR analyses were performed using *TwoSampleMR* R package^78^(R version 4.1.3).

#### Image analysis

Images were deconvolved using Imaris Clearview (Oxford Instruments) and an AMD Radeon Pro W5500 GPU with 8GB GDDR6. Image analysis was performed in ImageJ (NIH) using corrected total cell fluorescence (CTCF), which is the integrated density corrected for the product of cell area and mean background fluorescence.^79,80^ Brightness and contrast were linearly adjusted across the entire image for presentation purposes, and applied equally between all states under examination.

#### Transcriptomics analysis

Quantification data for all published scRNA-seq datasets were kindly provided by Leon Van Gurp.^30^ In brief, pseudo-counts were normalized for each data set using Seurat,^81^ and the cell identity was assigned based on the requirement for hormone gene expression to be in the top 1% expressed genes in each cell using Aucell.^82^ Quantification for published FACS sorted α and β cells were obtained from GEO database repository under Arda et al.^33^ The raw read files for each cell type were merged, trimmed and the transcripts were quantified using Kallisto^83^ or aligned and quantified as previously described,^84^ with similar results.

For Uniform Manifold Approximation Projection (UMAP) plots, datasets were downloaded from GEO: GSE150724,^30^ GEO: GSE84133^38^ and GEO: GSE114297.^39^ GEO: GSE84133^38^ contained a single donor with type 2 diabetes, excluded here. Seurat R package was used to filter, cluster, and identify cell types. Cells were filtered if they had less than 500 unique genes and if more than 20% of their UMI counts were of mitochondrial origin. Only moderate filtering of mitochondrial DNA was conducted because of the nature of the cells. After filtering, raw UMI counts were normalized by scaling to 10,000 followed by log2-transformation. Normalized gene expression was used to find the top 2000 most variable genes. These genes were ranked based on the number of samples in which they were deemed a top variable gene, scaled to generate z-scores and used to run Principal component analysis (PCA). The first 50 principal components were used to define anchors between datasets using Reciprocal PCA (RPCA). RPCA was run with Donor 1 from^30^ as a reference. Subsequently, PCA was run on the integrated data and a neighborhood graph built using 20 principal components. Clustering was carried out using a resolution of 0.2 and visualized in 2D space (Uniform Manifold Approximation Plot) for cluster identification. Cells clustered according to their cell type, as marked by their expression of INS, GCG and SST. Most cells in the original clusters had ambient levels of the non-identifying hormone, which might reflect cross-contamination during FACs.^85^ However, there were cells with low expression of the cluster-identifying hormone, and cells with high expression of the non-identifying hormones. These cells were excluded on the premise that they represent doublets, or misclustered cells. After cell type identification, raw counts from each cell were aggregated (summed) according to cell type and donor for ‘pseudobulk’ representation. Markers used for cell type identification are listed in Table S6.

### QUANTIFICATION AND STATISTICAL ANALYSIS

GraphPad Prism 9 (version 9.2.0) was used for all statistical analyses. Data distribution was assessed using the D’Agostino-Pearson normality test. Pairwise comparisons were made using unpaired t test, with Welch’s correction where standard deviation was non-equal between groups. Multiple interactions were determined using one-way ANOVA or two-way ANOVA, with Sidak’s post-hoc test, and adjusted for repeated measures where relevant. For non-parametric data, pairwise comparisons were made using Mann-Whitney test, and multiple interactions determined using Kruskal-Wallis test, with Dunn’s post-hoc test. Individual datapoints are shown in bar graphs. Unless otherwise stated in the figure legend, all error bars represent mean ± S.E.M. and a p value less than 0.05 was considered significant: *p < 0.05; **p < 0.01; ***p < 0.001; ****p < 0.0001.

## Supplementary Material

1

2

3

4

## Figures and Tables

**KEY RESOURCES TABLE:** 

REAGENT or RESOURCE	SOURCE	IDENTIFIER

Antibodies		

Mouse anti-LDH	Santa Cruz Biotechnology	Cat# sc-133123; RRID:AB_2134964
Rabbit anti-LDHB	Sigma-Aldrich	Cat# HPA019007; RRID:AB_2670008
Guinea pig anti-PDX1	Abcam	Cat# ab47308; RRID:AB_777178
Guinea pig anti-insulin	Abcam	Cat# ab7842; RRID: AB_306130
Mouse monoclonal anti-glucagon	Sigma-Aldrich	Cat# G2654; RRID:AB_259852
Rabbit anti mouse biotinylated	DAKO (OEM Polyclonal Antibodies)	Cat# E0413; https://www.agilent.com/en/oem-polyclonal-antibodies
Donkey anti rabbit biotinylated	NOVEX	Cat# A16039; RRID: AB_2534713
Goat anti-guinea pig Alexa Fluor 568	Thermo Fisher Scientific	Cat# A-11075; RRID: AB_141954
Goat anti-guinea pig Alexa Fluor 647	Thermo Fisher Scientific	Cat# A-21450; RRID: AB_141882
Goat anti-mouse pig Alexa Fluor 568	Thermo Fisher Scientific	Cat# A-11004; RRID: AB_2534072

Chemicals, peptides, and recombinant proteins		

SERVA NB8 collagenase	Amsbio	Cat# 17456.01
D-Glucose-^13^C_6_	Sigma-Aldrich	Cat# 389374
HardSet mounting medium with DAPI	VECTASHIELD	Cat# H-1500
Streptavidin-FITC	Sigma-Aldrich	Cat# S-3762
Fluo8	AAT Bioquest	Cat# 21082-AAT
AXKO-0046	MedChemExpres	Cat# HY-147216
Galloflavin	Tocris	Cat# 4795
Exendin4	Tocris	Cat# 1933

Critical commercial assays		

Lumit Insulin Immunoassay	Promega	CS3037A01
DeadEnd^™^ Fluorometric TUNEL System	Promega	G3250

Deposited data		

Islet scRNA-seq data	Van Gurp et al.^30^	GEO: GSE150724
FACS sorted α and β cell transcriptomic data	Arda et al.^33^	GEO: GSE79469
Pancreas scRNA-seq data	Baron et al.^38^	GEO: GSE84133
Islet scRNA-seq data	Xin et al.^39^	GEO: GSE114297

Experimental models: Cell lines		

EndoC-βH1	Human Cell Design	RRID:CVCL_IS72
EndoC-βH5	Human Cell Design	N/A

Experimental models: Organisms/strains		

Human cadaveric Islets	San Raffaele Hospital in Milan University of Lille and the Center Hospitalier Régional Universitaire de Lille University of Alberta, IsletCore University of Oxford, Oxford Consortium for Islet Transplantation	https://ecit.dri-sanraffaele.org/ https://www.bcell.org/adi-isletcore.html
CD-1 IGS mice	Charles River	Strain Code 022
C57BL/6	Charles River	Strain Code 027

Recombinant DNA		

β-cell specific Laconic Lactate biosensor	San Martín et al., 2013^43^; Sdao et al., 2021^44^	N/A
β-cell specific Perceval-HR ATP/ADP biosensor	Tantama et al., 2013^61^; Lewandowski et al., 2020^11^	N/A

Software and algorithms		

GraphPad Prism 9.5.1	GraphPad software	https://www.graphpad.com/features
Fiji	NIH	https://imagej.net/software/fiji/
MddNMR v2.5	Kazimierczuk & Orekhov, 2011^62^	http://mddnmr.spektrino.com/
NMRPipe v9.2	Delaglio et al., 1995^63^	https://spin.niddk.nih.gov/bax/software/NMRPipe/NMRPipe.html
MetaboLab	Ludwig & Günther, 2011^64^	https://www.ludwiglab.org/software-development
Imaris Clearview	Oxford Instruments	https://imaris.oxinst.com/products/clearview-gpu-deconvolution
R Studio	R Project	https://www.r-project.org/
CellSens	Olympus	https://www.olympus-lifescience.com/en/software/cellsens/
GPower 3.1.9.7	Heinrich Heine University Düsseldorf	https://www.psychologie.hhu.de/arbeitsgruppen/allgemeine-psychologie-und-arbeitspsychologie/gpower
MetaMorph 7.10.5	Molecular Devices	https://www.moleculardevices.com/products/cellular-imaging-systems/high-content-analysis/metamorph-microscopy

Other		

Olympus Fluoview FV3000	Evident	RRID:SCR_017015
Crest X-Light V2	CretsOptics	https://crestoptics.com/x-light-v2/
Leica SP8	Leica	RRID:SCR_018169
Agilent 8890 GC	Agilent	RRID:SCR_019459
Agilent 5977B GC	Agilent	RRID:SCR_019420
Bruker *Neo* 800 MHz NMR	Bruker	https://www.bruker.com/en/products-and-solutions/mr/nmr/ascend-nmr-magnets.html
High fat diet	Research Diets	Cat# D12492

## Highlights

- Human and rodent islets generate lactate following glucose stimulation
- β cells specifically express LDHB, which acts to limit lactate generation
- LDHB inhibition leads to inappropriate insulin release at low glucose
- *LHDB cis*-EQTLs associate with fasting glucose in humans
