# Supplementary material for: LDHB contributes to the regulation of lactate levels and basal insulin secretion in human pancreatic β cells
Source: Cell Rep. Author manuscript; Available in PMC 2024 Jun 10. (PMC11164428; doi:10.1016/j.celrep.2024.114047)
Supplement: 1 [file NIHMS1988705-supplement-1.pdf]

Cell Reports, Volume *43*

Supplemental information

LDHB contributes to the regulation of lactate levels and basal insulin secretion in human pancreatic β cells

Federica Cuozzo, Katrina Viloria, Ali H. Shilleh, Daniela Nasteska, Charlotte Frazer-Morris, Jason Tong, Zicong Jiao, Adam Boufersaoui, Bryan Marzullo, Daniel B. Rosoff, Hannah R. Smith, Caroline Bonner, Julie Kerr-Conte, Francois Pattou, Rita Nano, Lorenzo Piemonti, Paul R.V. Johnson, Rebecca Spiers, Jennie Roberts, Gareth G. Lavery, Anne Clark, Carlo D.L. Ceresa, David W. Ray, Leanne Hodson, Amy P. Davies, Guy A. Rutter, Masaya Oshima, Raphaël Scharfmann, Matthew J. Merrins, Ildem Akerman, Daniel A. Tennant, Christian Ludwig, and David J. Hodson

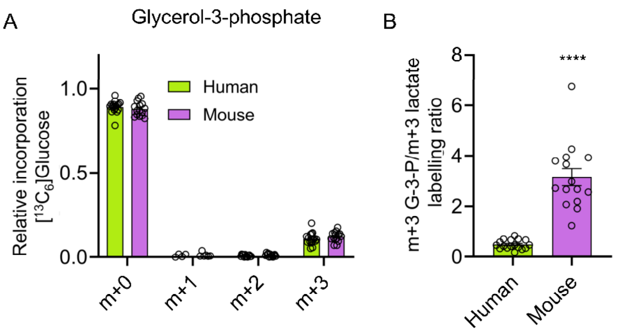

Figure S1: Glycolytic contribution to the accumulation of lactate. Related to Figure 1. A) MID analysis showing similar incorporation of ^{13}C from $^{13}C_6$ glucose into m+0 and m+3 glycerol-3-phosphate in human *versus* mouse islets (two-way ANOVA and Sidak's post-hoc test). **B)** Labelling ratio of fully labelled G-3-P over fully-labelled lactate is significantly higher in mouse than human islets (unpaired t-test). For all data, n = 17 islet preparations, 9 donors and n = 15 islet preparations, 8 animals. Bar graphs show individual datapoints and mean ± SEM. ****P<0.0001.

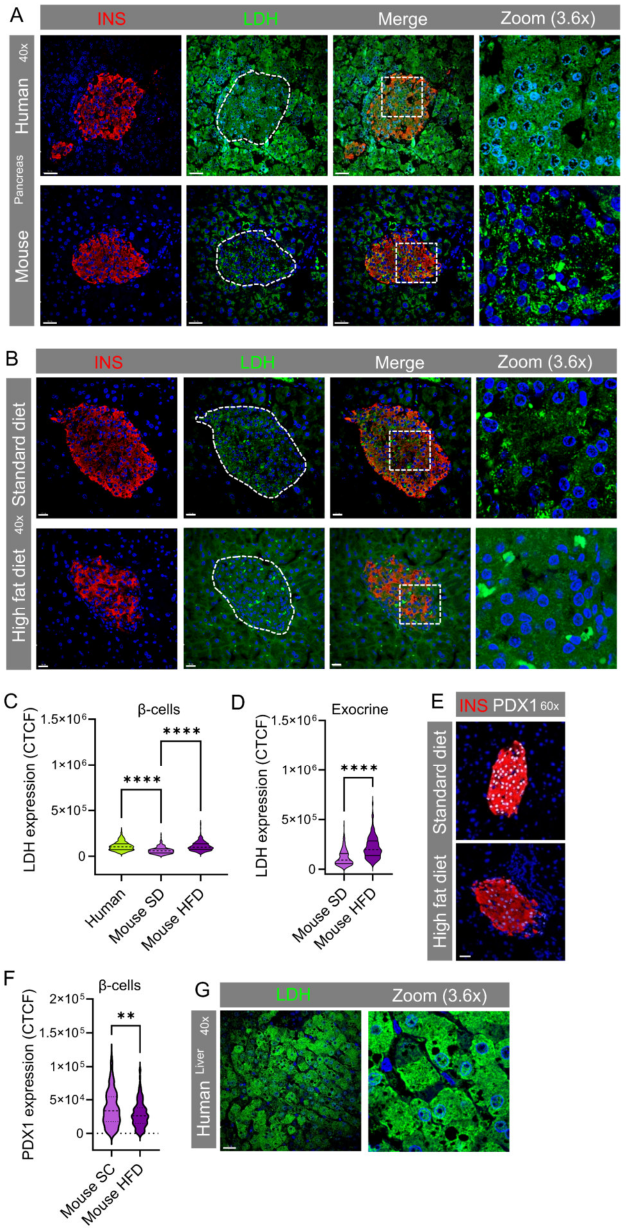

Figure S2: Human islets express LDHA, LDHB and LDHC protein. Related to Figure 3. A) LDH expression, assessed using an antibody with cross-reactivity against LDHA, LDHB and LDHC, is higher in human β cells compared to mouse β cells. **B)** LDH expression increases in mouse exocrine tissue (outside dotted line) and β cells following 8-12 weeks of high fat diet (HFD) feeding versus age-matched standard diet (SD) controls. **C)** Corrected total cell fluorescence (CTCF) quantification of LDH protein expression in human β cells (n = 240 cells, 3 donors), SD mouse β-cells (n = 220 cells, 3 animals), and HFD mouse β cells (n = 250 cells, 3 animals) (Kruskal-Wallis test, Dunn's post-hoc test). **D)** As for C), but showing LDH immunoreactivity in the exocrine pancreas of mice fed either SD or HFD (n = 230-250 cells, 3 animals) (Mann-Whitney test). **E)** PDX1 expression is decreased in β cells of mice fed HFD for 8 weeks versus SD controls (n = 240 cells, n = 3 animals). **F)** CTCF quantification of PDX1 protein expression in SD and HFD mouse β-cells (n = 240 cells, 3 animals) (Mann-Whitney test). **G)** Immunostaining showing strong LDH expression in human liver from donors with non-

alcoholic steatohepatitis (n = 240 cells, n = 3 donors) (Mann-Whitney test). Representative images are shown. Scale bar = 30 μm. Bar graphs show individual datapoints and mean ± SEM. Violin plot shows median and interquartile range. AU = arbitrary unit. **$P<0.01$; ****$P<0.0001$.

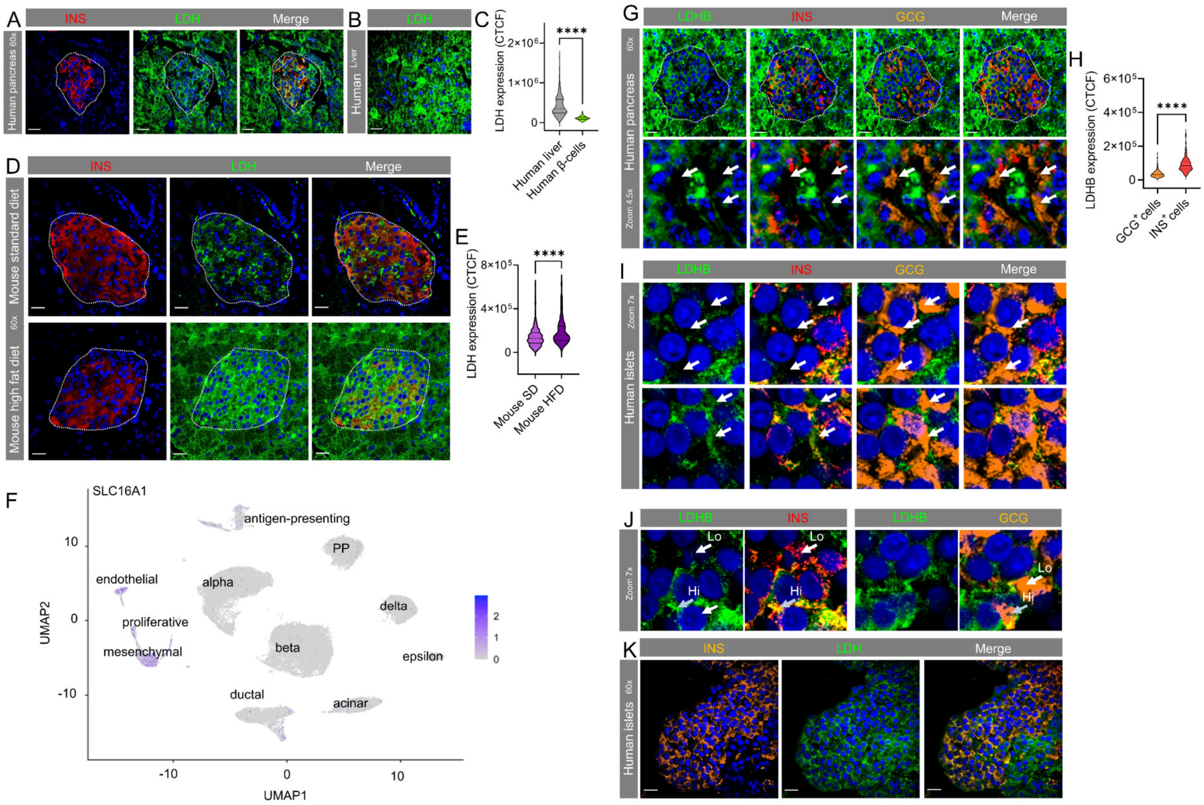

Figure S3: Re-quantification of LDH and LDHB in human and mouse pancreas sections at higher magnification, and scRNA-seq analysis of *SLC16A1* expression in human β-cells. Related to Figure 3 and Figure S2. A-C) New sections were cut from the same donor pancreata (A) and livers (B) as used in Figure S2A and G, before repeat immunostaining for LDH and quantification with a 60x, 1.30 NA objective. Note the much higher LDH expression in human liver versus β-cells (C), identified by insulin positivity (n = 300 cells, 3 donors). **D, E)** Pancreata from mice fed either standard diet or high fat diet were re-imaged with a 60x, 1.30 NA objective, showing similar results to those in Figure S2B and C (n = 279-295 cells, 3 animals). **F)** UMAP plot showing *SLC16A1* raw counts clustered according to cell type (data taken from n = 18 donors in[1-3]). **G, H**) New sections were cut from the same donor pancreata (G) as used in Figure 3G-J before repeat immunostaining for LDHB and quantification with a 60x, 1.30 NA objective. Confirming the results at 40x, LHDB is expressed at very low to undetectable levels in α-cells (H) (n= 300 cells, 3 donors). **I)** Zoom-in (top panel) of isolated human islet in Figure 3K showing that LDHB protein expression strongly co-localizes with insulin (INS), but not glucagon (GCG). Zoom-in (bottom panel) of isolated human islet in Figure 3K showing a small subpopulation of GCG+ cells with detectable LDHB staining. **J)** Zoom-in from isolated human islet in Figure 3K, but showing classification of LDHB Hi and Lo cells for GCG and INS. **K)** LDH (LDHA + LDHB) immunostaining in isolated human islets, showing a similar pattern and intensity to that seen in pancreatic slices (Figure S2A). All data were analyzed using Mann-Whitney test. Scale bar = 30 µm. Violin plot shows median and interquartile range. ****P<0.0001.

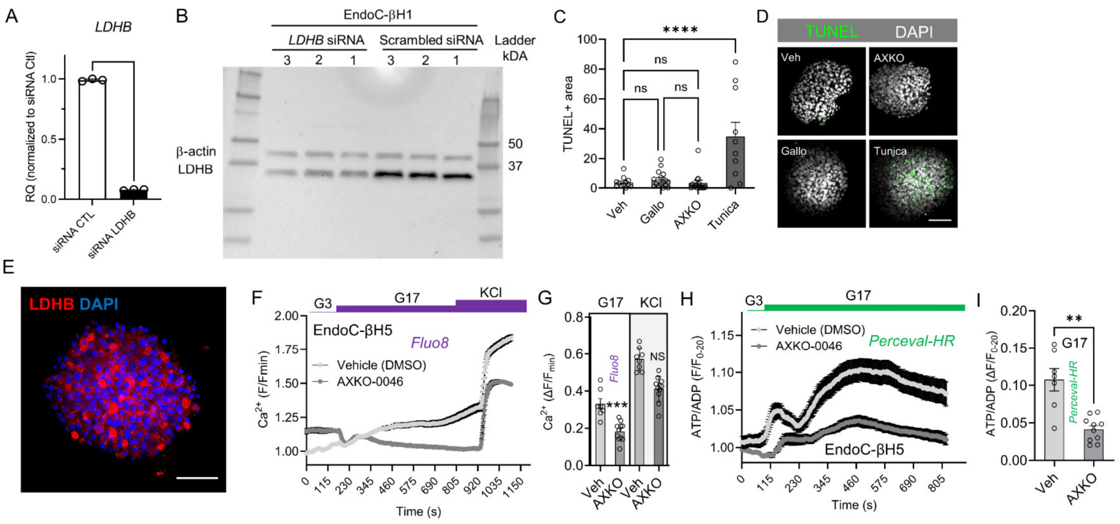

Figure S4: LDHB studies in EndoC-βH1 and EndoC-βH5 cells, and apoptosis studies in human islets. Related to Figure 4. A) qPCR analysis of *LDHB* expression in RNA extracted from EndoC-βH1 cells transfected with siRNA CTL (scrambled) and siRNA *LDHB*. Values were normalized to a house keeping gene, *PPIA*, followed by normalization to siRNA CTL. **B)** Protein lysates were extracted from EndoC-βH1 cells transfected with LDHB and CTL siRNA. Expected band for LDHB is ~35kDa. β-actin is used as loading control with an expected band of ~45kDa. (n=3 is used for both experimental groups; full uncropped blot is shown). **C, D)** AXKO-0046 (10 µM) and galloflavin (10 µM) do not affect apoptosis versus vehicle (DMSO) (Tunica; 20 µg/ml tunicamycin is positive control) (n = 10-14 islets, 3 donors) (one-way ANOVA, Sidak's post-hoc test) (scale bar = 62 µm). **E)** EndoC-βH5 spheroid immunostained for LDHB (n = 5 spheroids) (scale bar 53 µm). **F, G)** Vehicle-treated EndoC-βH5 spheroids respond to 17 mM glucose with increases in cytosolic Ca^{2+}, an effect suppressed by pre-incubation with 100 nM AXKO-0046, as shown by mean ± SEM traces (F) and summary bar graph (G) (n = 11-13 spheroids) (Mann-Whitney test). **H, I)** As for F, G), but ATP/ADP ratios measured using Perceval-HR (n = 7-10 spheroids) (Mann-Whitney test). G17, 17 mM glucose; G3, 3 mM glucose. Traces show mean ± SEM. Bar graphs show individual datapoints and mean ± SEM. AXKO, AXKO-0046; G3, 3 mM glucose; G17, 17 mM glucose. NS, non-significant; **P<0.01; ****P<0.0001.

Table S2: Human islet donor characteristics. Related to Figures 1-4 and Figure S1-4. BMI, body mass index. IFG, impaired fasting glucose.

Unique identifier	Age group (years)	Gender	BMI (Kg/m^2)	Glycemia (mmol/L)* HbA1C (%)	History of diabetes**	Islet purity (%)	Islet culture duration (h)	Country of origin
**HP1404**	50-55	♂	29.4	7.8 mmol/L	N/A	80	18	Italy
**HP1406**	60-65	♂	26.1	N/A	N/A	90	96	Italy
**HP1408**	55-60	♀	19.0	N/A	N/A	90	18	Italy
**HP1416**	60-65	♂	31.1	N/A	No but IFG	75	20	Italy
**HP1419**	55-60	♂	22.8	7.3 mmol/L	N/A	90	18	Italy
**HP1431**	60-65	♀	26.9	8.0 mmol/L	N/A	90	18	Italy
**HI1117**	45-50	♂	24.0	5.4%	N/A	80	N/A	France
**HI1120**	50-55	♂	29.5	5.7%	N/A	90	N/A	France
**HI1121**	60-65	♂	32	5.5%	N/A	90	N/A	France
**R496**	65-70	♀	22.3	5.6%	N/A	95	N/A	Canada
**HP2337**	35-40	♀	29.71	N/A	N/A	80	N/A	UK
**HP2338**	50-55	♀	30.11	N/A	N/A	70	N/A	UK
**H1236**	45-50	♂	29.2	5.7%	N/A	90	N/A	France
**HP2339**	35-40	♂	30.39	N/A	N/A	70	N/A	UK
**R511**	45-50	♀	19.2	5.9%	N/A	90	N/A	Canada
**R512**	60-65	♂	34.7	5.9%	N/A	90	N/A	Canada
**R513**	60-65	♀	26.4	5.3%	N/A	40	N/A	Canada
**R521**	25-30	♀	31	5.1%	N/A	70	N/A	Canada
**HP2402**	45-50	♀	25.95	35 mmol/L	N/A	65	N/A	UK
**HP2403**	50-55	♂	27.78	N/A	N/A	75	N/A	UK
**R526**	35-40	♂	36.5	6.1%	N/A	40	N/A	Canada
**ID1489**	60-65	♀	22.2	N/A	N/A	60	N/A	Italy

Table S5: Human pancreas and liver donor characteristics. Related to Figure 3, Figure S2 and Figure S3. BMI, body mass index. ND, non-diabetic. NASH, non-alcoholic steatohepatitis.

Sample ID	Disease	Tissue	Age	BMI (Kg/m^2)	Sex	Cause of death
43-D	ND	Adult pancreas	78	-	F	Myocardial infarction
137-D	ND	Adult pancreas	67	-	M	Ischaemic heart disease
77-C	ND	Adult pancreas	56	-	F	Cerebral aneurysm
LT6 T0	NASH	Adult liver	54	28.4		Stroke
LT7 T12	NASH	Adult liver	41	35		Intracranial haemorrhage
LT8 T48	NASH	Adult liver	69	24.8		Intracranial haemorrhage
HP18-42	ND	Adult isolated islets	58	30	F	Intracranial haemorrhage
HP18-54	ND	Adult isolated islets	49	26.37	F	Cardiac arrest
HP18-32	ND	Adult isolated islets	48	32.3	F	Intracranial haemorrhage

Table S6: Markers used for cell type selection in UMAP plots. Related to Figure 3 and Figure S3.

Cell type	Markers
Endothelial	CD34, PECAM1, CD93
Proliferative	MKI67
Mesenchymal	TNFAIP6, THY1
Acinar	CTRC, PRSS1
Ductal	CFTR, KRT19, SOX9
Antigen Presenting	HLA-DRA, CXCL8, HLA-DRB1, CD83
Beta	PDX1, INS, IAPP
Delta	SST, PDX1
Alpha	GCG
Epsilon	GHRL
PP	PYY
Non-endocrine cells	CHGA-, NEUROD1-
Endocrine	CHGA+, NEUROD1+

References

1. van Gurp, L., Fodoulian, L., Oropeza, D., Furuyama, K., Bru-Tari, E., Vu, A.N., Kaddis, J.S., Rodriguez, I., Thorel, F., and Herrera, P.L. (2022). Generation of human islet cell type-specific identity genesets. Nat Commun *13*, 2020. 10.1038/s41467-022-29588-8.
2. Baron, M., Veres, A., Wolock, S.L., Faust, A.L., Gaujoux, R., Vetere, A., Ryu, J.H., Wagner, B.K., Shen-Orr, S.S., Klein, A.M., et al. (2016). A Single-Cell Transcriptomic Map of the Human and Mouse Pancreas Reveals Inter- and Intra-cell Population Structure. Cell Syst *3*, 346-360 e344. 10.1016/j.cels.2016.08.011.
3. Xin, Y., Kim, J., Okamoto, H., Ni, M., Wei, Y., Adler, C., Murphy, A.J., Yancopoulos, G.D., Lin, C., and Gromada, J. (2016). RNA Sequencing of Single Human Islet Cells Reveals Type 2 Diabetes Genes. Cell Metab. *24*, 608-615. 10.1016/j.cmet.2016.08.018.
